# Physiological analysis and transcriptome sequencing reveal the effects of combined cold and drought on tomato leaf

**DOI:** 10.1186/s12870-019-1982-9

**Published:** 2019-08-27

**Authors:** Rong Zhou, Xiaqing Yu, Tongmin Zhao, Carl-Otto Ottosen, Eva Rosenqvist, Zhen Wu

**Affiliations:** 1 Department of Food Science, Aarhus University, Årslev, Denmark; 2 Laboratory for Genetic Improvement of High Efficiency Horticultural Crops in Jiangsu Province, Institute of Vegetable Crop, Jiangsu Province Academy of Agricultural Sciences, Nanjing, Jiangsu China; 3 National Key Laboratory of Crop Genetics and Germplasm Enhancement, Nanjing Agricultural University, Nanjing, Jiangsu China; 4 Department of Plant and Environmental Sciences, University of Copenhagen, Taastrup, Denmark

**Keywords:** Tomato, Combined stress, Physiological response, RNA sequencing

## Abstract

**Background:**

Co-occurrence of cold and drought stress can alter the response of plants at morphological, physiological and molecular levels, which finally affect crop production, more than individual stress. Understanding the responses of crop to combined stress is necessary to improve tolerance and maintain crop production especially in the field where combined stress frequently occurs. We aimed to clarify the underlying leaf physiological and molecular mechanisms of tomato by imposing combining cold and drought on one popular tomato cultivar ‘Jinlingmeiyu’ as an example.

**Results:**

The physiological and genetic responses were identified in tomatoes after 42 h exposure to control, cold, drought and combined treatments. As compared with control, water loss rate at the three stresses including cold, drought and combined stress significantly decreased until 40 min after taking samples from the plants. The content of H_2_O_2_, zeatin riboside (ZR) and melatonin in all stress treatments were significantly higher than the control. Drought stress alone and combined stress induced the accumulation of abscisic acid (ABA) and auxin (IAA) as compared with control. The individual cold and combined stress significantly decreased the maximum quantum efficiency of PSII (F_v_/F_m_), quantum yield of PSII (F_q_^′^/F_m_^′^) and electron transport rate (ETR). In total, 7141, 1850 and 7841 genes were involved in the stress response to cold, drought and their combination. Functional analysis of the stress-inducible genes provided more insights concerning the complex regulatory mechanisms that were involved in combined stress. The expression level of 12 genes were validated by quantitative real-time PCR (qRT-PCR).

**Conclusions:**

We found that the expression of stress-specific genes changed with physiological variation, indicating the close crosstalk between physiological and genetic response especially under combined stress. This study provides new knowledge on the complex regulatory mechanism genes in tomato (‘Jinlingmeiyu’) leaf to abiotic stresses.

**Electronic supplementary material:**

The online version of this article (10.1186/s12870-019-1982-9) contains supplementary material, which is available to authorized users.

## One sentence summary

Combination of cold and drought is a new state stress with cold as a dominant factor in tomato.

## Background

Plants suffer low temperature and water shortage in cold- and drought-prone regions in the world [1–3]. Cold stress has been reported to negatively affect plant growth and development in various crops, such as rice [4], wheat [5] and tomato [6, 7]. Likewise, drought stress is a natural challenge for crops including tomato with an adverse effect on production [1, 8, 9]. Due to the rapid and dynamic global environmental changes, plants grown under the field conditions more and more frequently face a combination of various abiotic stresses [10], such as cold and drought. Studies involving cold and drought stress normally address the stress individually, while only few studies combine cold and drought [10, 11].

Tomato (*Solanum lycopersicum* L.) is an economically important crop worldwide, which is sensitive to a series of abiotic stresses, especially extreme temperature and drought. Cold and drought have been reported to decrease the tomato production, the damage of which was aggravated as a consequence of global climate change [12, 13]. Simultaneous cold and drought negatively affected crop growth and hampered productivity [11, 14], leading to economy loss. Tomato seedlings could easily suffer low temperature and water deficit in the beginning of spring and the late of winter in many regions of China. For instance, the promotion of cultivar ‘Jinlingmeiyu’ in Jiangsu Province were restricted due to the cold and drought stress. Therefore, it is crucial to determine how tomatoes such as ‘Jinlingmeiyu’ respond to combined cold and drought stresses.

Relative water content (RWC) is a quantitative indicator of plant water status [6]. Cold stress (3 °C for 16 h) and drought stress (without irrigation for 5 days) induced a significant drop in the RWC of tomato [6, 15], which was correlated with the water loss. Higher leaf RWC of tomato under cold stress was promoted by exogenous H_2_O_2_ pretreatment [6]. Tomato plants can maintain acquired tolerance though increasing NADPH oxidase-mediated H_2_O_2_ to temperature stress such as heat stress [16]. Drought stress resulted in an increase in reactive oxygen species (ROS) triggering a response by signal transduction pathways with H_2_O_2_ as secondary messenger, which was closely related to ABA (abscisic acid) [17]. Phytohormones such as ABA, IAA (auxin), GA_3_ (gibberellin), ZR (zeatin riboside) and melatonin played a dynamic and important role in increasing the tolerance of plants to abiotic stress [11]. For instance, both cold and drought stress induced ABA accumulation in plants [17, 18]. Previous studies clearly demonstrated the significance of IAA in cold−/drought-mediated plant growth, regulated by polar deployment and trafficking of auxin carriers [19, 20]. The reduction of GA biosynthesis in plant under abiotic stress contributed to growth restraint mediated by DELLA proteins [21]. On the contrary, enhanced cold tolerance together with high ZR content was observed in transgenic tall fescue containing the *Agrobacterium tumefaciens ipt* gene [22]. Melatonin is naturally an antioxidant in plants that can efficiently remove ROS [23]. In addition, due to the high sensitivity of the photosynthesis to abiotic stress, lower net photosynthesis rate and decreased F_v_/F_m_ (maximum quantum efficiency of PSII) were observed in tomato under cold (3 ± 2 °C for 16 h) [7] and drought without irrigation for 5 days [15]. Generally, the plants under cold and drought stress share a number of common responses in the aspect of phytohormones being related to ROS, antioxidant response and signaling transduction. [11]. However, changes in phytohormones content of tomato under combined cold and drought as compared with individual stress remained unclear and how the changes affect tomato photosynthesis and growth need to clarify.

As a consequence of the increased efficiency of RNA sequencing (RNA-seq), transcriptome analysis has identified many genes involved in cold or drought response in plants, such as rice [24], maize [25] and tomato [26, 27]. Profound changes in the gene expression in plants were induced by cold [3] and drought [28]. For example, genes involved in signal transduction (e.g. mitogen-activated protein kinase or MAPK) and osmotic stress signal perception (e.g. MYB29) were both responsive to cold and drought conditions [11]. Furthermore, WRKY transcription factors has been reported to play important roles in response to abiotic stresses including cold and drought [29, 30]. Some of the genes were induced only by cold, while some genes are drought-inducible only [11]. A key characteristic of both cold and drought stress is the accumulation of ABA that can promote a series of plant response such as ABA-dependent gene expression [18, 31].

Crop physiology and genomics have provided useful knowledge to improve plant tolerance (Tuberosa & Salvi, 2006). To the best of our knowledge, the physiological and molecular response of tomatoes under combined cold and drought stress are scarce. This study focuses on the responsive mechanisms of tomato under cold, drought and their combination in relation to leaf water status, photosystem II (PSII) activity, antioxidants, phytohormones and key genes by transcriptome analyses. We aim to clarify the effect of cold, drought and their combination ranging from leaf physiological and molecular levels and expound the relationship between individual and combined stress response with tomato cultivar ‘Jinlingmeiyu’ as an example. Our hypotheses are that 1) combined cold and drought stress create a different stress response compared to individual stress with cold as a dominant stress factor; 2) combined cold and drought stress negatively affected tomato leaf physiological response regulated by genes involved in signal transduction and photosynthetic electron transport. This study provides overview responses of tomato ‘Jinlingmeiyu’ to combined stresses by integrating plant physiological and transcriptional approaches.

## Results

### Physiological responses

**Fig. 1:**
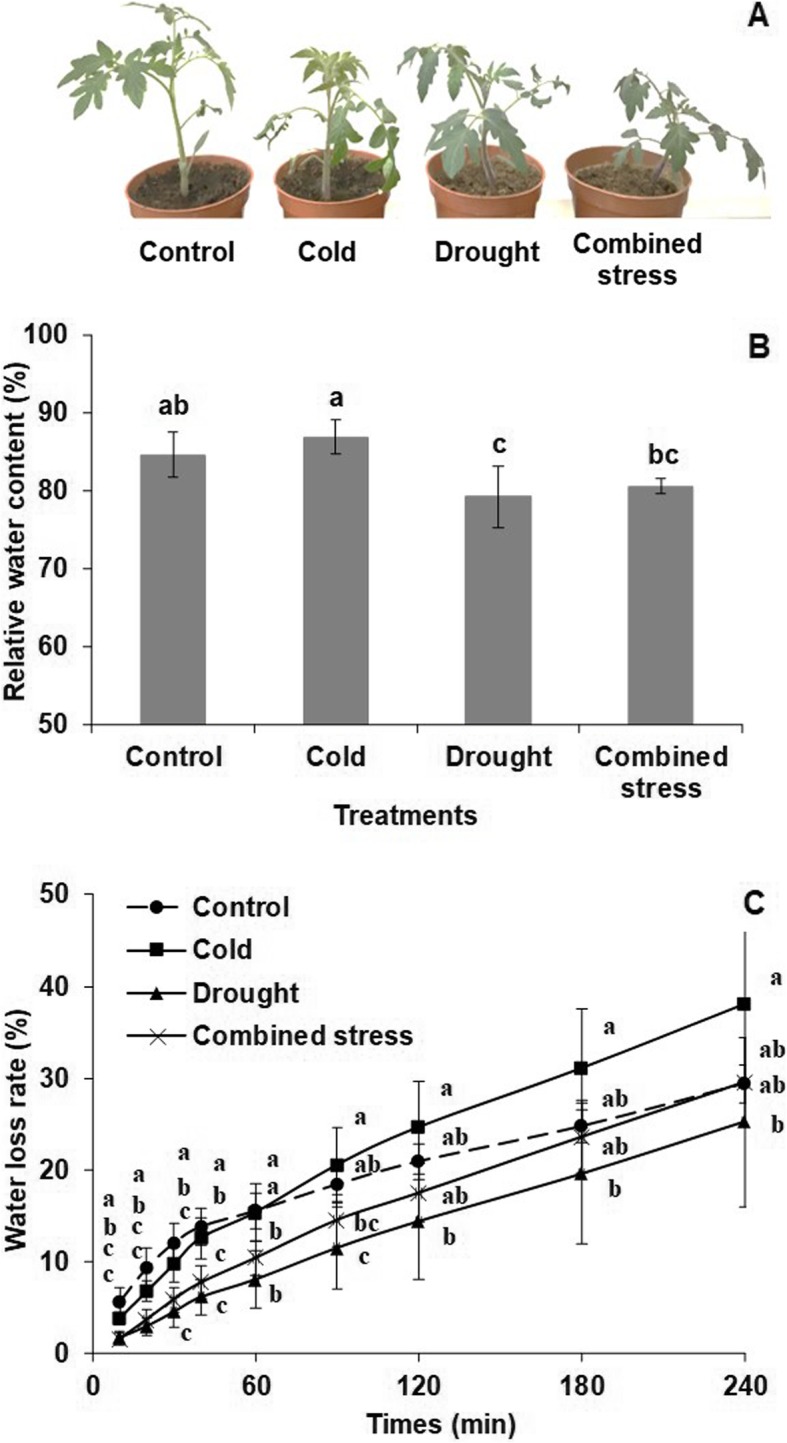
**a** Morphological performance, (**b**) relative water content and (**c**) water loss rate of tomato after 42 h exposure to control, cold, drought and combined stress. The data represents mean values ± SD (*n* = 4). Different letters indicate significant difference at *P* < 0.05

**Fig. 2:**
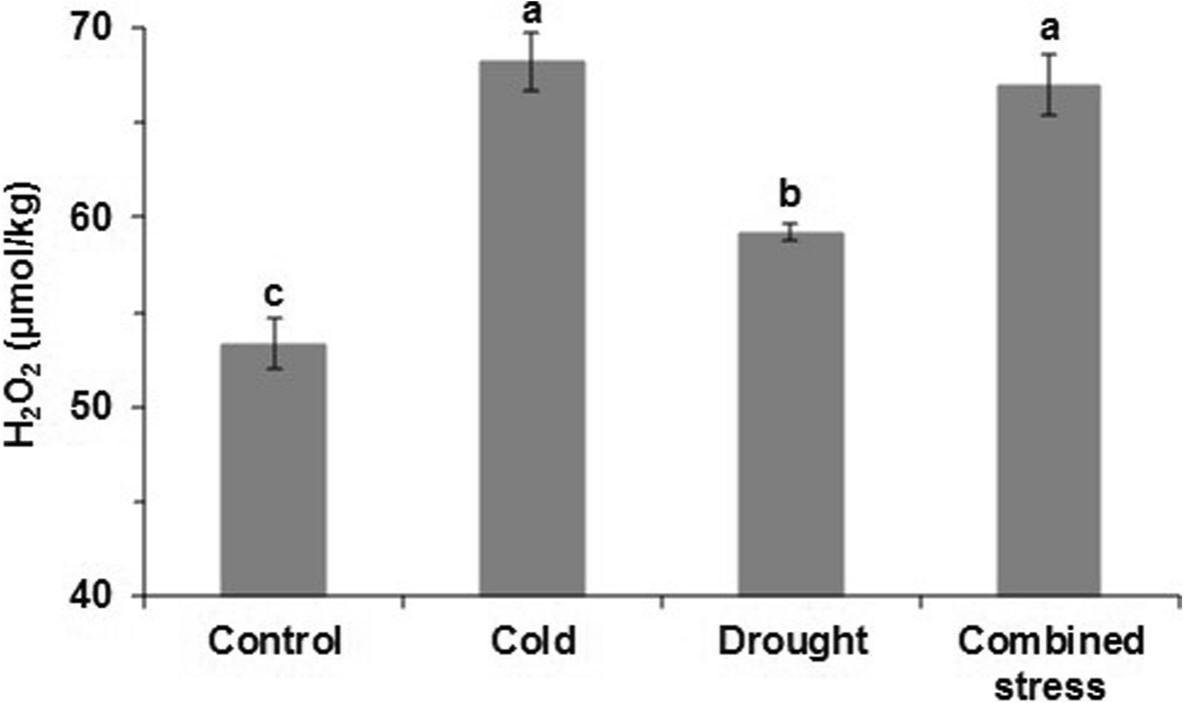
The H_2_O_2_ content in leaves of tomato at control, cold, drought and combined stress. The data represents mean values ± SD (*n* = 3). Different letters indicate significant difference at *P* < 0.05

Tomato exposed to the three stresses for 42 h showed slower growth than the control (Fig. 1a). The leaf RWC after drought stress was significantly lower than in the control, while that after cold stress was significantly higher than drought and combined stress (Fig. 1b). The water loss rate after all stress exposures was significantly lower than control from 10 min to 40 min after sampling, but this trend disappeared in the late stage (from 120 min to 240 min) (Fig. 1c). In addition, all stress treatments significantly increased the H_2_O_2_ content in the leaves with the highest H_2_O_2_ content induced by cold and combined stress (Fig. 2).

**Fig. 3:**
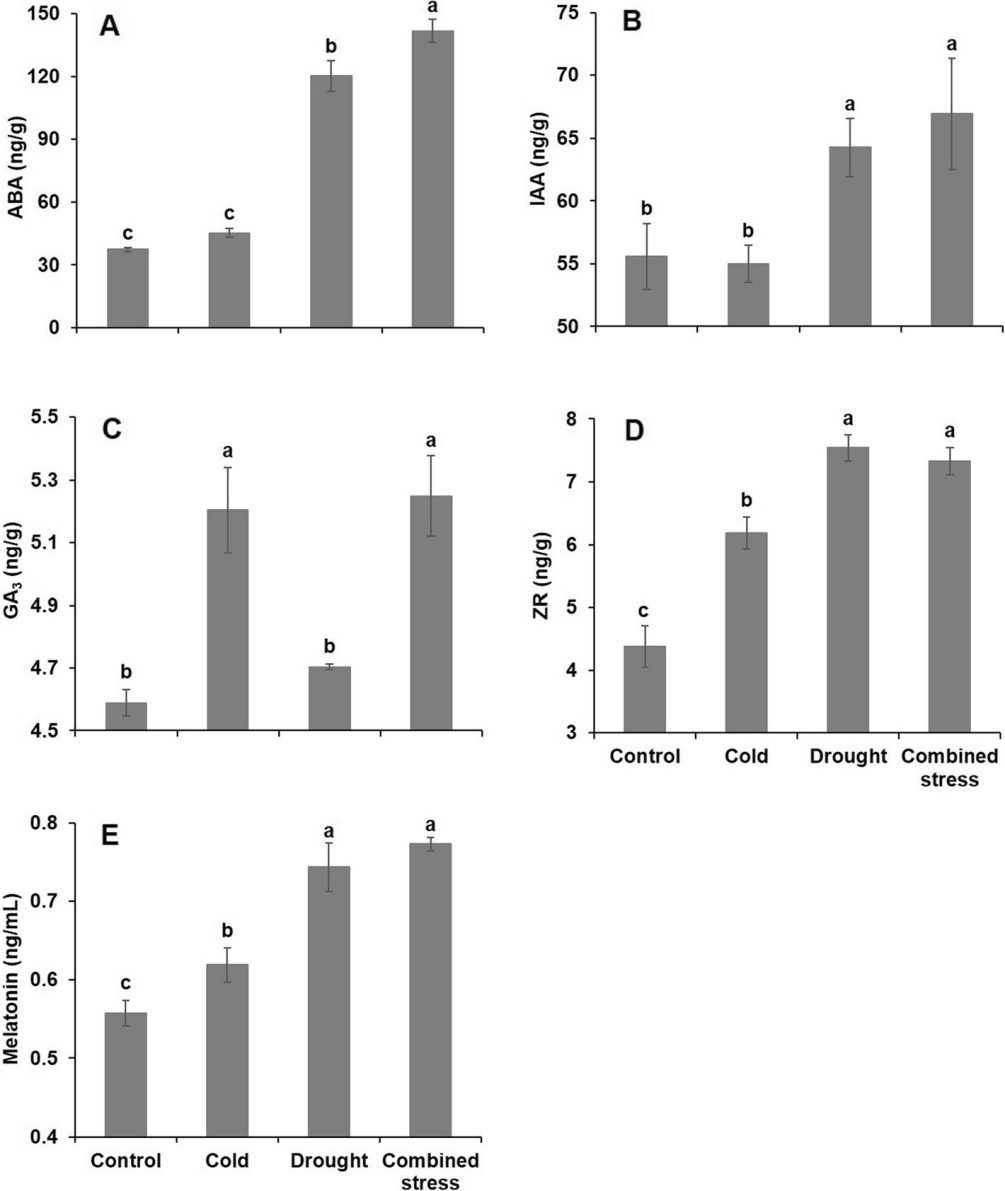
The content of (**a**) ABA (abscisic acid), (**b**) IAA (auxin), (**c**) GA_3_ (gibberellin), (**d**) ZR (zeatin riboside) and (**e**) melatonin in leaves of tomato after 42 h exposure to control, cold, drought and combined stress conditions. The data represents mean values ± SD (n = 3). Different letters indicate significant difference at *P* < 0.05

The phytohormones were regulated very differently by the various stress treatments. The content of ABA and IAA were only upregulated when the tomato plants were exposed to drought alone or combined with cold (Fig. 3a, b), and for ABA more after the combined stress than after single drought stress. The content of GA_3_ was only upregulated when exposed to cold alone and combination stress (Fig. 3c), while ZR and melatonin were upregulated by all stress treatments but more if drought was involved alone or in combination with cold (Fig. 3d, e).

**Fig. 4:**
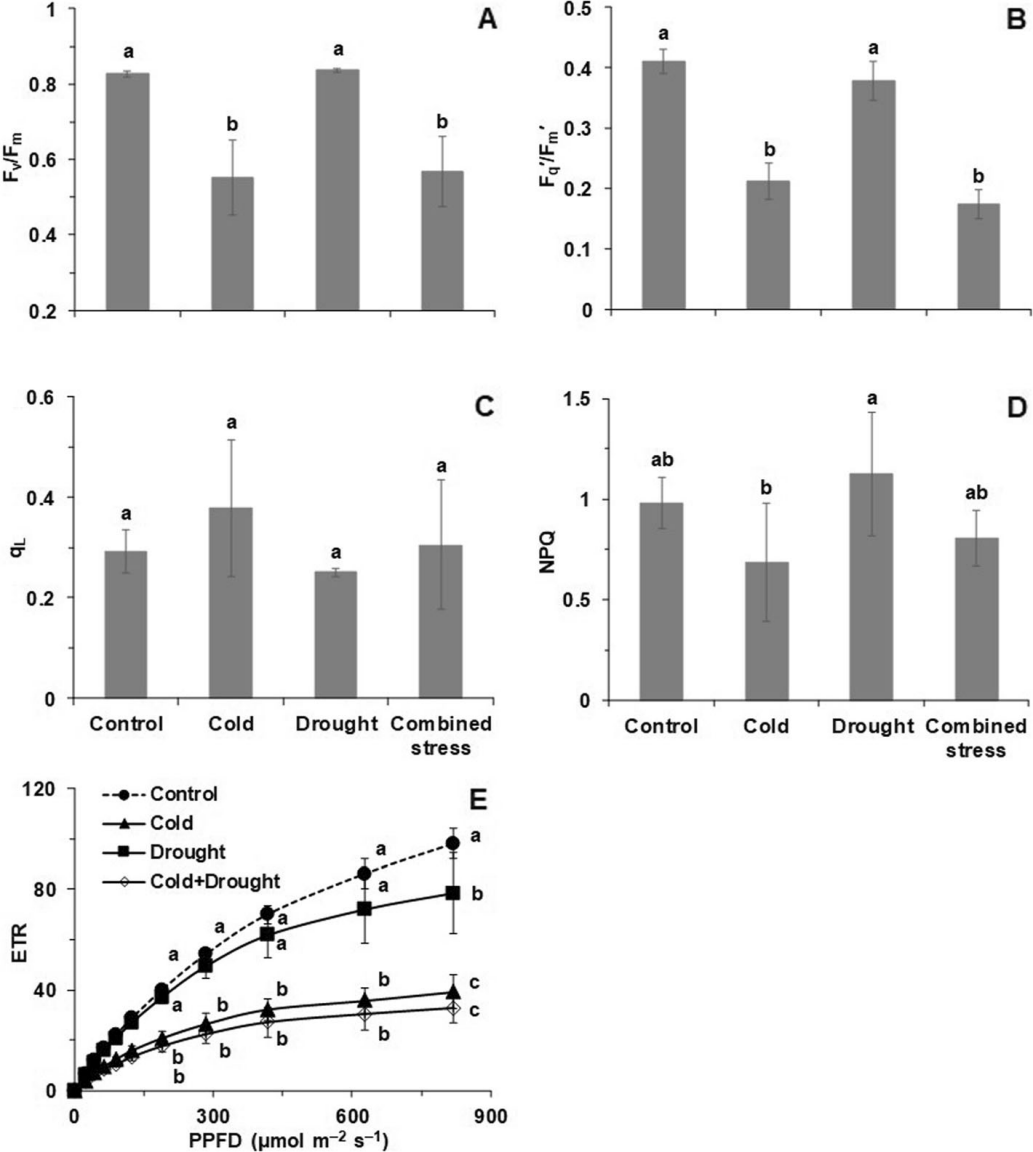
**a** The F_v_/F_m_ (maximum potential quantum efficiency of PSII), (**b**) F_q_^′^/F_m_^′^ (quantum yield of PSII), (**c**) q_L_ (fraction of open PSII centers), (**d**) NPQ (non-photochemical quenching) and (**e**) rapid light-response curves of ETR (electron transport rate) in leaves of tomato after 42 h exposure to control, cold, drought and combined stress conditions. The F_q_^′^/F_m_^′^, q_L_ and NPQ were measured at a PPFD of 285 μmol m^− 2^ s^− 1^, which was close to the growth PPFD of 300 μmol m^− 2^ s^− 1^. The data represents mean values ± SD (n = 4). Different letters indicate significant difference at *P* < 0.05. For ETR, the different letters were marked for light levels of 190, 285, 420, 630 and 822 μmol m^− 2^ s^− 1^

The F_v_/F_m_ and F_q_^′^/F_m_^′^ (quantum yield of PSII) after cold and combined stress were significantly lower than control and drought stress (Fig. 4a, b). The q_L_ (fraction of open PSII centers) showed the opposite pattern from NPQ (non-photochemical quenching) with cold increasing and drought decreasing q_L_ but here the variation prevented any establishment of significant differences (Fig. 4c). After 42 h stress, drought increased and cold decreased NPQ making the cold and drought treatments significantly different with the control and combined stress in between (Fig. 4d). The electron transport rate (ETR) in the cold and the combined stress treatments decreased significantly compared with control at PPFD (photosynthetic photon flux density) from 0 to 822 μmol m^− 2^ s^− 1^ (Fig. 4e). By comparison, in the drought treatment ETR was significantly lower than control only at the highest PPFD (Fig. 4e).

### Molecular responses

The four cDNA libraries of tomato treated by control, cold, drought and their combination generated 25,082,239, 29,815,250, 28,754,112 and 27,195,304 clean reads with 43.3, 43.0, 42.8 and 43.3% GC percentage, respectively. The respective Q_30_ percentage (sequencing error rates < 0.1%) was 92.9, 93.0, 92.5 and 91.9% in tomato in response to control, cold, drought and their combination, indicating that the sequencing data was qualified for further analysis. In total, there were 50,162,479, 59,630,499, 57,508,225 and 54,390,609 total reads identified in tomato in response to the four treatments (Additional file 3: Table S1). De novo assembly predicted 755 new genes that have not appeared in tomato genome database annotated by COG (Clusters of Orthologous Groups, http://www.ncbi.nlm.nih.gov/COG/), GO (Gene Ontology Consortium, http://www.geneontology.org/), KEGG (Kyoto Encyclopedia of Genes and Genomes, http://www.genome.jp/kegg/), KOG (Eukaryotic Orthologous Groups, http://www.ncbi.nlm.nih.gov/KOG/), Pfam (Homologous Protein Family, http://pfam.xfam.org/), Swissprot (A manually annotated, non-redundant protein sequence database, http://www.uniprot.org/), eggNOG (evolutionary genealogy of genes: Non-supervised Orthologous Groups, http://eggnog.embl.de/) and nr (non-redundant protein sequence database, ftp://ftp.ncbi.nih.gov/blast/db/) (Additional file 1: Figure S1). The eggNOG function classification of consensus sequence showed that the function of the majority of the genes remained unknown and many genes played roles in the section of ‘L’ (replication, recombination and repair), ‘Q’ (secondary metabolites biosynthesis, transport and catabolism) and ‘T’ (signal transduction mechanisms) (Additional file 1: Figure S1).

**Fig. 5:**
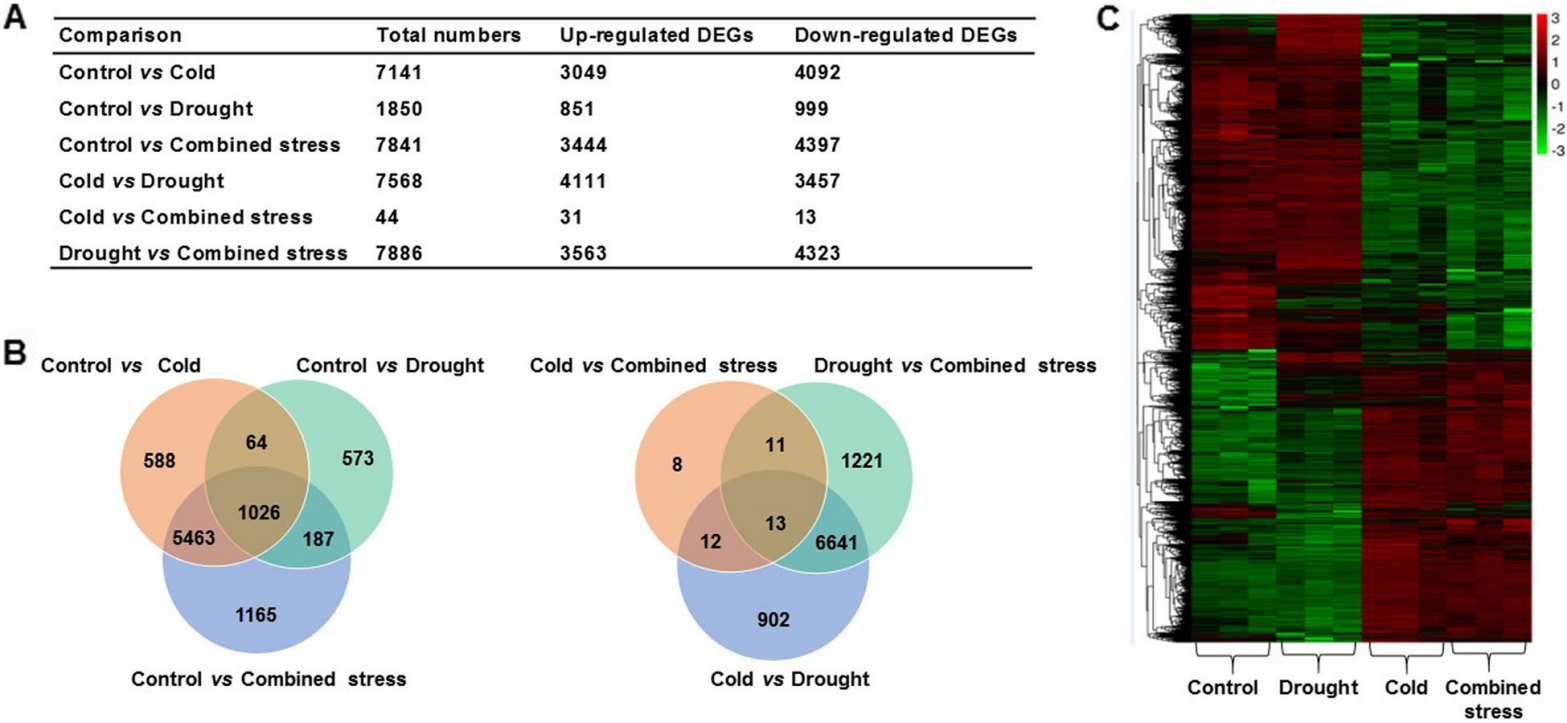
**a** The DEGs (differentially expressed genes) in tomato at different stress conditions, (**b**) veen diagram of the DEGs in tomato for different comparisons and (**c**) volcano plot in tomato in control and after 42 h exposure to cold, drought and combined stress conditions

RNA-seq provided an overview of genes differentially expressed in tomato during the different stresses (Fig. 5). Compared with control, 7141, 1850 and 7841 genes were significantly differently expressed in tomato after cold, drought and combined stress, respectively (Fig. 5a). Combined stress induced significantly different expression levels of 44 and 7886 genes in comparison with individual cold and drought, respectively (Fig. 5a). Among the significantly DEGs (differentially expressed genes), 1165 genes existed only in control vs combined stress as compared with control vs cold and control vs drought (Fig. 5b). Overall, the expression pattern of the genes in tomato during cold were similar to combined stress (Fig. 5c).

**Fig. 6:**
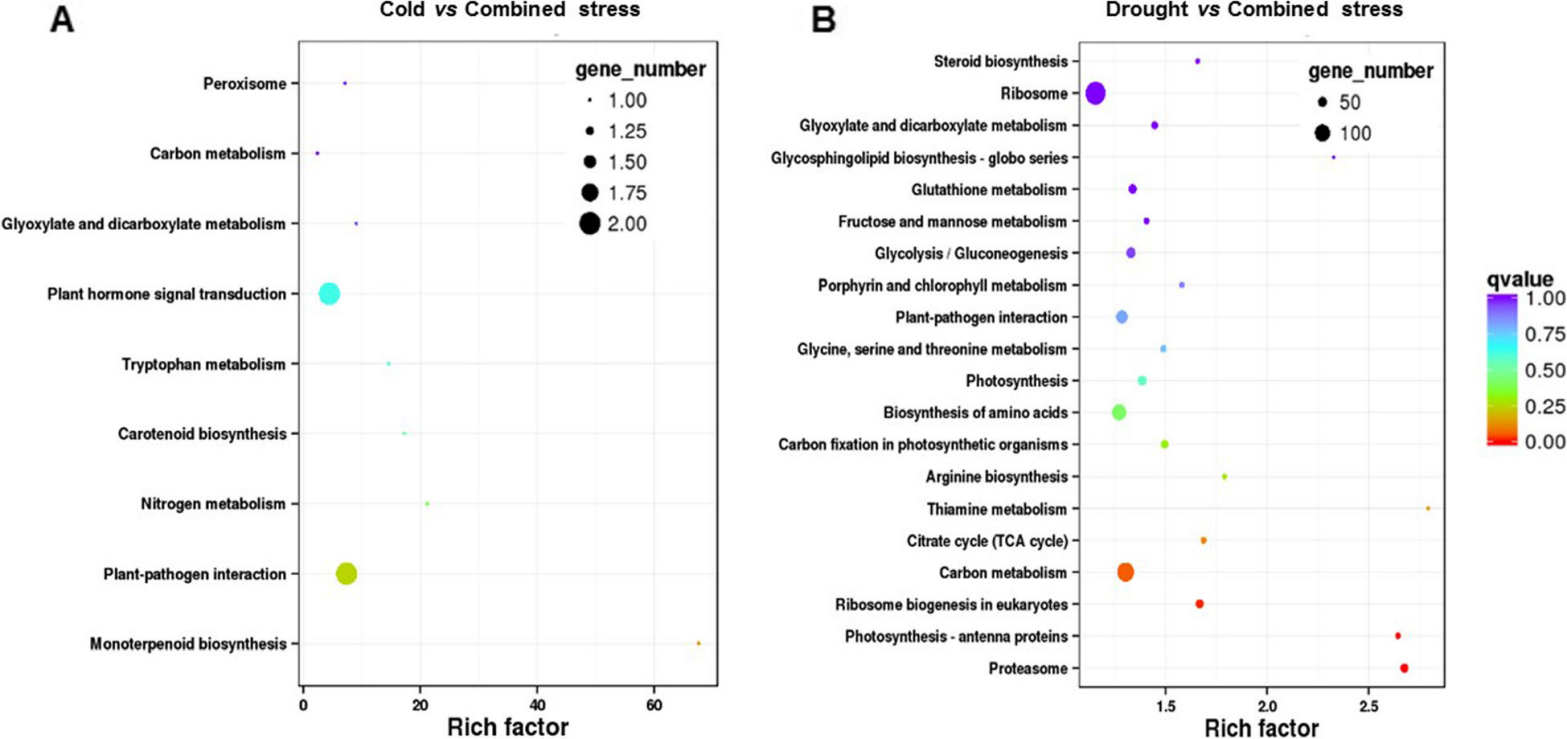
The KEGG (Kyoto Encyclopedia of Genes and Genomes, http://www.genome.jp/kegg) pathway enrichment analysis of the DEGs (differentially expressed genes) between (**a**) cold vs combined stress and (**b**) drought vs combined stress

The genes with significantly different levels in tomato after combined stress had a role in carbon metabolism, biosynthesis of amino acids, plant-pathogen interaction and so on (Additional file 2: Figure S2). When compared to cold stress, genes expressed in response to combined stress played roles in phytohormone signal transduction, plant-pathogen interaction etc. (Fig. 6a). The genes responsive to combined stress differed from that of drought with a role in ribosomes, carbon metabolism, biosynthesis of amino acids, plant-pathogen interaction, glycolysis/gluconeogenesis etc. (Fig. 6b).

**Fig. 7:**
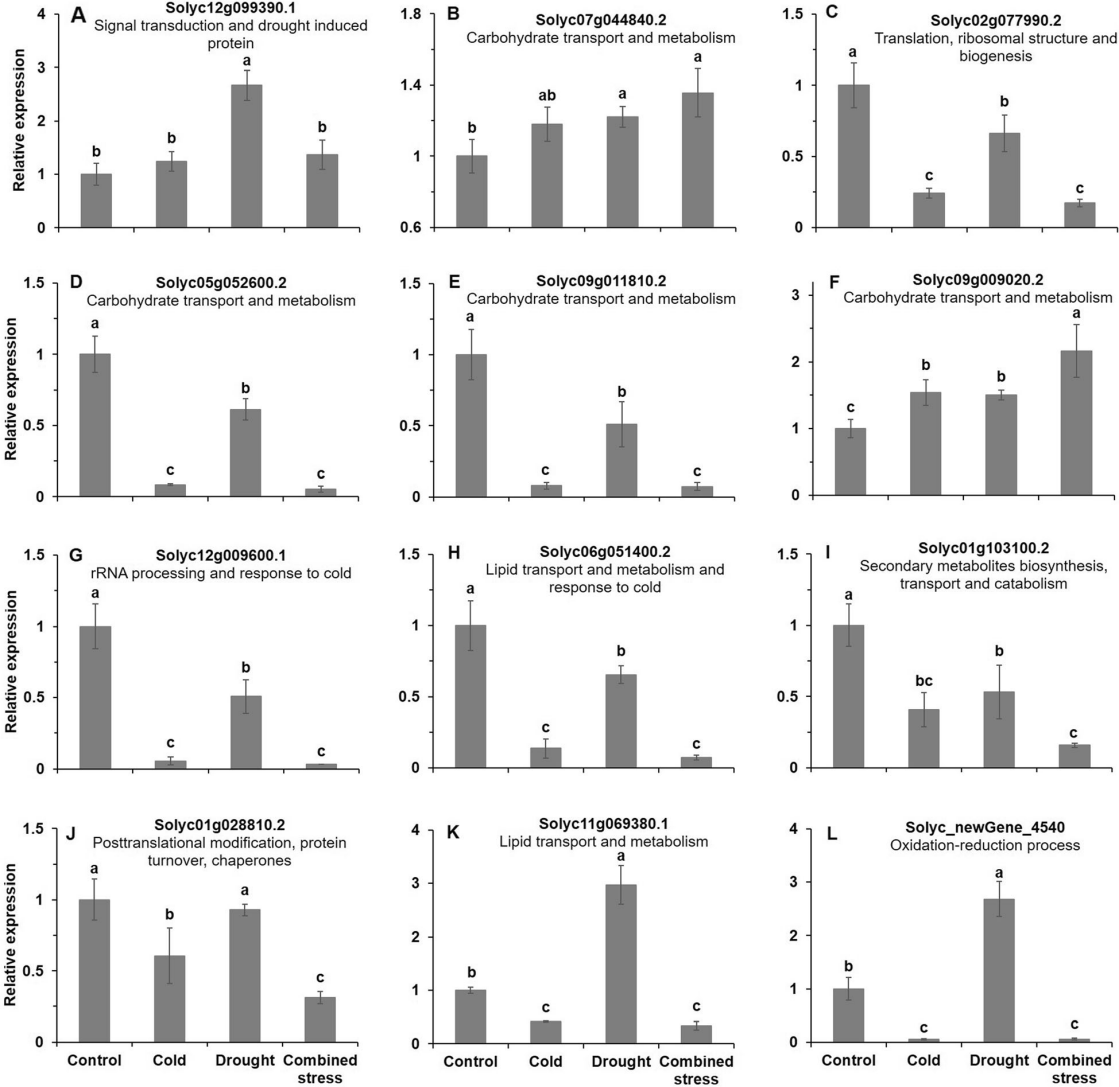
The expression level of the selected 12 genes by qRT-PCR (quantitative real-time PCR) validation. Solyc_newGene_4540 was the abbreviation of Solanum_lycopersicum_newGene_4540. The primary function of each genes were shown below the gene name. The data represents mean values ± SD (n = 3). Different letters indicate significant difference at *P* < 0.05

The expression levels of 12 genes with significantly changed expression levels based on RNA-seq were detected by qRT-PCR (quantitative real-time PCR validation). The drought stress significantly increased the expression level of Solyc12g099390.1 in comparison with control (Fig. 7a). The expression level of Solyc07g044840.2 in tomato after drought and combined stress was significantly higher than control (Fig. 7b). The cold, drought and combined stress significantly decreased the expression level of Solyc02g077990.2, Solyc05g052600.2, Solyc09g011810.2, Solyc12g009600.1, Solyc06g051400.2 and Solyc01g103100.2, but significantly increased the expression level of Solyc09g009020.2 (Fig. 7c-i). Compared with the control, the expression level of Solyc01g028810.2, Solyc11g069380.1 and Solanum_lycopersicum_newGene_4540 (Solyc_newGene_4540) in tomato dropped significantly after cold and combined stress, while the expression level of Solyc11g069380.1 and Solyc_newGene_4540 in tomato increased significantly after drought stress (Fig. 7j-l). The primary functions of the 12 verified genes were signal transduction, carbohydrate transport and metabolism, translation as well as posttranslational modification responding to drought and cold (Fig. 7). The details of the 12 genes were shown Additional file 3: Table S2.

## Discussion

Plants respond to cold and drought stress through a series of biochemical, physiological and genetic processes [11, 32]. Molecular and genomic studies have shown that the genes with a wide variety of functions are induced by cold and drought stresses [32]. Understanding the mechanisms of physiology and genes involved in stress signaling networks and stress tolerance is the key for crop improvement and production [3, 11].

### The physiological responses were closely related to key genes in tomato during stress

The common phenotypic symptoms of low temperature and water shortage in plants are leaf wilting, stunted growth and even death during the exposure [3, 17]. In accordance with our previous studies [15, 33], drought stress decreased the RWC and water loss rate in tomato (Fig. 1). Plants exposed to cold stress often show water-stress symptoms due to cold-induced inhibition of water uptake and loss [6, 17]. Thereby, leaf wilting and curling in tomato in response to three stresses were correlated with low RWC and decreased water loss rate, especially in the early stage after sampling.

In addition to changes in water status, increased H_2_O_2_ content was observed in tomato responding to three stresses (Fig. 2), indicating that low temperature and water shortage conditions caused ROS generation. With the chosen stress levels, the chilling temperature created more H_2_O_2_ than the drought. We found that the expression level of genes being involved in the ROS response were significantly changed by the three stresses (Additional file 3: Table S3). For example, as compared with control, the expression of Solyc02g086050.2 and Solyc12g070270.1 significantly decreased by 1.76 and 1.48, while that of Solyc02g078360.2 significantly increased by 1.29 in tomato leaf under cold stress. Similarly, the three genes involved in ROS responding to cold stress showed the same trend in expression levels to combined stress. Besides, the expression level of Solyc10g044550.1 was down-regulated (− 1.61) in tomato leaf under drought condition in comparison with control. The H_2_O_2_ may be part of the signal to turn on the stress-responsive genes in tomato under cold and/or drought stress as indicated by Cruz de Carvalho (2008) [17] and Yadav (2011) [3]. The H_2_O_2_ can act as an oxidative burst signal, activating the stress defense system in plants [34]. In this study, 829, 175 and 907 genes with roles in signal transduction mechanisms were induced by cold, drought and combined stress, respectively (Additional file 3: Table S4). Numerous transcription-factor genes were stress-inducible and different transcriptional regulatory mechanisms were induced by cold and/or drought stress in signal transduction pathways [35, 36]. Accordingly, in this study, cold, drought and combined stress induced transcription factors such as members of the basic-domain leucine zipper (bZIP) family, the MYB family and the WRKY family. These transcription factors can regulate many stress-inducible genes and establish gene networks in response to various stress conditions in tomato.

Moreover, phytohormones are crucial components in various signal pathways [37], and for instance, ABA can stimulate the synthesis of H_2_O_2_ in guard cell and in turn H_2_O_2_ mediated ABA-dependent stomatal closure [38]. A significant crosstalk among the drought and ABA responses have been shown since more than half of the drought-inducible genes are also induced by ABA treatments [36]. Taking account into the increased ABA content in tomato after drought and combined stress (Fig. 3), it is suggested that there was a positive relationship between the water deficiency and ABA enhancement. A similar response of ABA and IAA (growth promoting hormone) was observed in tomato in all stresses, which was consistent with the result in cold-shock treated wheat [39]. Reduction of GA content and signaling adversely affected plant growth under abiotic stress such as cold [21]. However, it is opposite with our finding that cold induced the GA_3_ accumulation regardless of dourght stress, which could be due to the effect of other hormonal responses. ZR contents in ipt-transgenic lines with enhanced cold tolerance were 65–400% higher than non-transformed control [22], suggesting that the studied tomato actively responded to the stress conditions by increasing ZR content. Previous study found that melatonin was involved in the regulation of ROS and ABA in *Malus* species when exposed to drought [38]. Increased melatonin content in tomato during drought stress help to alleviate the ROS damage due to comparatively lower H_2_O_2_ content as compared with cold and combined stress. Therefore, it is suggested that inducing a signal transduction such as the production of H_2_O_2_ and phytohormones is a key mechanism for plants to respond to abiotic stress. ABA synthesis is one of the quickest responses of plants under abiotic stress that trigger the expression of ABA-inducible gene [40]. We found that 120, 43 and 125 genes playing roles in the phytohormone signal transduction were significantly regulated by cold, drought and combined stress, respectively (Additional file 3: Table S5). This can be partly explained by the significant changes in phytohormone contents in tomato under the stress conditions (Fig. 3).

The tomato exhibited significant variation in PSII activity especially after exposure to cold and combined stress. According to Baker and Rosenqvist (2004) [41], as expected, drought alone did not affect neither F_v_/F_m_ nor the fluorescence quenching parameters measured at the growth PPFD (Fig. 4). Any stress that involved 10 °C temperature, however, significantly decreased F_v_/F_m_ and the operating efficiency, F_q_^′^/F_m_^′^, at the growth PPFD. Neither the oxidation state of PSII, q_L_, and the light controlled heat dissipation in the antenna, NPQ, were significantly affected at the growth PPFD (Fig. 4). However, in light of the increasing difference between the treatments with increasing PPFD seen in the rapid light response curve of ETR, it implied that the tendencies seen at intermediate PPFD become significantly different when approaching light saturation. Meanwhile, we found that 36, four and 38 genes with significantly different expression levels in tomato under cold, drought and combined stress, respectively, were involved in the photosynthetic electron transport (Additional file 3: Table S6). Such physiological and genetic responses in tomato under cold or combined stress in turn lead to ROS generation and oxidative damage if protective mechanisms could not dissipate the excessive energy, which partially explained why tomato under cold and combined stress showed higher H_2_O_2_ content than drought.

### Combined cold and drought was a new state of stress condition with cold as a dominant factor

Deng et al. (2012) found that the physiological changes induced by drought are quite different from that induced by cold [42]. This was confirmed in our study by the physiological parameters such as RWC, water loss rate, H_2_O_2_ content, phytohormone content and chlorophyll fluorescence. Sales et al. (2013) found that drought stress alone (11 days without irrigation) severely affected ETR in sugarcane [43]. We found that ETR was more affected by cold and combined stress than drought, since an increase in photorespiration might have protected ETR from damage caused by drought. More importantly, in most cases here, the physiological responses were similar between cold and combined stress, suggesting the dominant role of low temperature when cold was combined with drought. The irrigation was stopped three days before temperature stress since water deficit is a gradual process that took time to happen. The possible reason for the dominant role of cold was that the response of tomato to drought was slow due to gradual water deficit, while the response of tomato to low temperature was very fast.

**Fig. 8:**
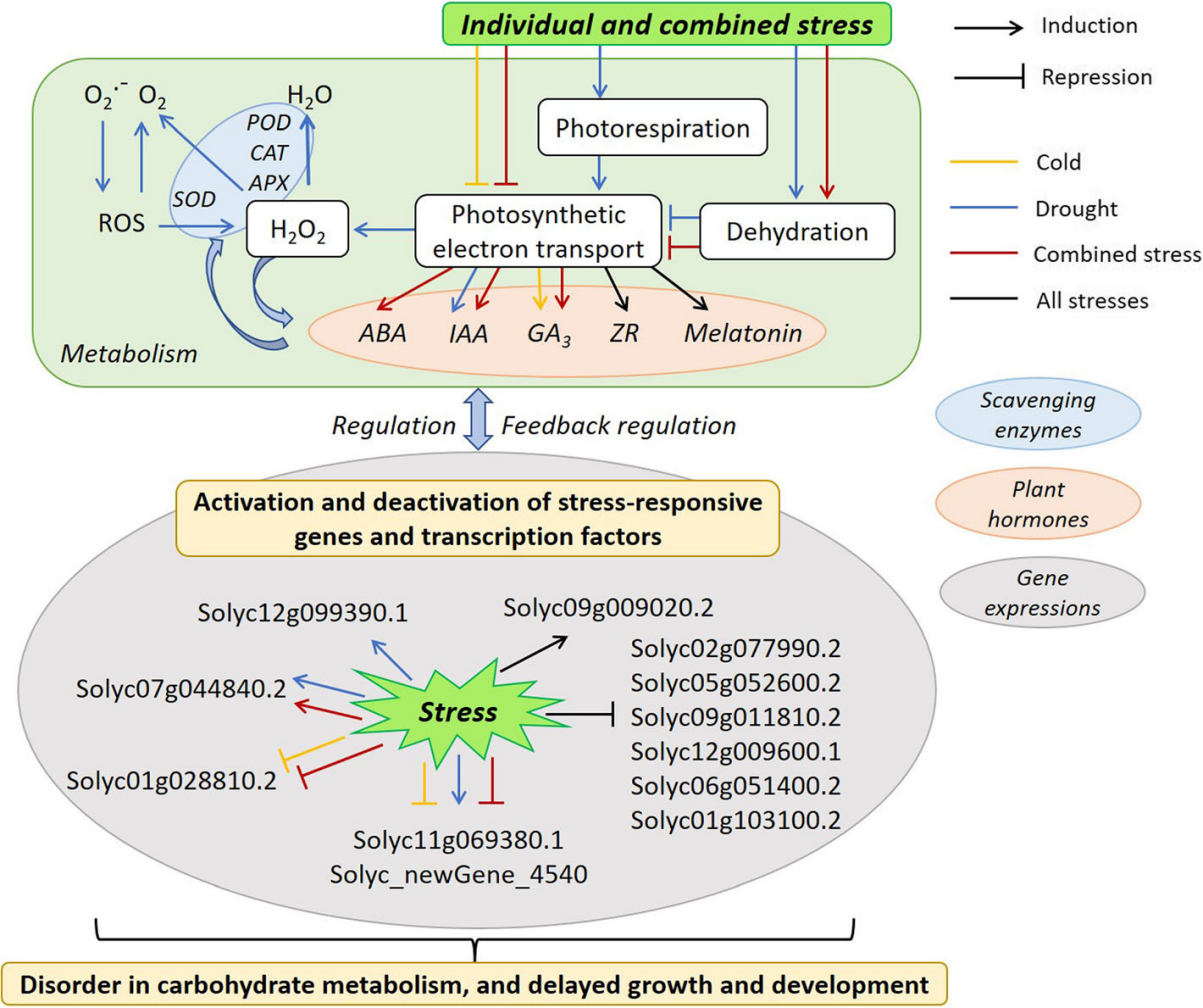
How tomato respond to individual and combined cold and drought in our case. The yellow, blue and red line indicated that the response to individual cold, individual drought and combined stress, respectively. The black line indicated that the responses happened after all the three stress conditions

Only 10% of the drought-inducible genes were also induced by cold [36], being consistent with our results that a few genes were shared in tomato by individual cold and drought condition. Apart from the physiological response, 4111 and 3457 genes were up- and down- regulated by drought as compared with cold (Fig. 5), indicating different molecular strategies in their reaction to the two stresses. Cold and drought induced common stress-inducible genes, while one of the stresses specifically induced some genes [44]. By contrast, a few genes with different expression levels between cold and combined stress were also found (Fig. 5). For example, the genes involved in ROS response showed the similar pattern in expression level when the plants were exposed to cold and combined stress, but exhibited a different pattern under drought and combined stress (Additional file 3: Table S3). As compared with individual cold and drought, there were three and 852 specific genes involved in signal transduction mechanisms responding to combined stress, respectively (Additional file 3: Table S4). This explained why a few genes played roles in tomato under combined stress in comparison with cold (Fig. 6). In our previous study, drought stress played a dominant role when combined with heat stress [15]. Obviously, it is not the same story when drought was combined with cold as indicated by the complex physiological and molecular networks responding to combined cold and drought. Combined cold and drought stress induced dehydration and declined tomato photosynthetic electron transport followed by ROS metabolism disorder and increased hormone level, during which the activation of stress-responsive genes and transcription factors happened (Fig. 8). However, the photosynthetic electron transport was less affected after drought than cold and combined stress due to an increasing photorespiration, which was accompanied with lower H_2_O_2_ and GA_3_ content (Fig. 8). The fact that the overlapping genes in response to individual and combined stress in tomato as shown in Fig. 8 highlights their importance in enhancing the tolerance of plants to abiotic stress. By comparison, specific genes responding to individual and combined stress, respectively, indicated that combined stress is a new state of condition with the activation of specifically responsive genes (Fig. 8).

## Conclusions

In general, combining cold and drought stress was a new state of stress condition rather than a simple addition of individual stresses on tomato ‘Jinlingmeiyu’ seedlings. The crosstalk between leaf physiology and gene expressions involved in ROS, phytohormone, signal transduction, photosynthetic electron transport and carbohydrate transport in tomato at individual and combined stress induced the disorder in carbohydrate metabolism, which contributed to delayed growth and development in plant under abiotic stresses (Fig. 8). This study provides us insights into the leaf physiological and molecular mechanisms of tolerance and adaption to cold, drought and their combination in plants with tomato ‘Jinlingmeiyu’ as an example.

## Methods

### Plant material and growth condition

Single seeds of the popular commercial tomato cultivar ‘Jinlingmeiyu’ bred by Jiangsu Province Academy of Agricultural Sciences were sown in plug trays. The seedlings were grown at 26/18 °C (14 h/10 h, day/night) and 50–60% relative humidity in climate chambers (RDN-560E-4, Dongnan Instrument Co, Ltd., Ningbo, China). The light intensity was 300 μmol m^− 2^ s^− 1^ PPFD provided by white LED light (Dongnan Instrument Co, Ltd., Ningbo, China) at the level of the plants. The plants were irrigated by water every five days after sowing and irrigated by nutrient solution every three days 15 days after sowing. The nutrient solution was made up based on the Japanese Garden test formula [Ca(NO_3_)_2_•4H_2_O, 945 mg L^− 1^; KNO_3_, 809 mg L^− 1^; NH_4_H_2_PO_4_, 153 mg L^− 1^; MgSO_4_•7H_2_O, 493 mg L^− 1^; FeSO_4_•7H_2_O, 13.9 mg L^− 1^; Na_2_-EDTA, 18.6 mg L^− 1^; H_3_BO_3_, 2.86 mg L^− 1^; MnSO_4_•4H_2_O, 2.13 mg L^− 1^; ZnSO_4_•7H_2_O, 0.22 mg L^− 1^; CuSO_4_•5H_2_O, 0.08 mg L^− 1^; (NH_4_)_6_Mo_7_O_24_•4H_2_O, 0.02 mg L^− 1^]. The 21-days-old uniform plants were transferred to plastic pots (11 cm diameter, 9 cm height) with one plant per pot. Both the plug trays and plastic pots were filled with sphagnum substrate (Pindstrup, Denmark). After three days, we stopped irrigating half of the seedlings. The 27-day-old irrigated plants were treated at continuous 26 °C and 10 °C for 42 h at 300 μmol m^− 2^ s^− 1^ as control and cold stress treated plants, respectively. The 27-day-old non-irrigated plants were treated at 26 °C and 10 °C for 42 h at 300 μmol m^− 2^ s^− 1^ as drought and combined stress treated plants, respectively.

### Leaf relative water content and water loss rate

The last fully expanded leaf from the top was cut from the plants after 42 h of the treatments with four replications. The fresh weight (FW) of the leaf was measured immediately. The leaf was immersed in dd-H_2_O and incubated at room temperature for four hours. Turgid weight (TW) of the leaf was measured after taking the leaf out form dd-H_2_O and blotting water from the leaf surface. Dry weight (DW) of the leaf was measured by drying the leaf at 80 °C for 24 h. The relative water content (RWC) of the leaf in % was calculated as RWC = [(FW - DW)/(TW - DW)] _*_ 100. The FW of the leaf immediately after cutting (0 min), 10 min, 20 min, 30 min, 40 min, 60 min, 90 min, 2 h, 3 h and 4 h after cutting were measured at room temperature. Water loss rate of the leaf x min after cutting in % = [(FW_0 min_ - FW_x min_)/FW_0 min_] _*_ 100.

### Leaf H_2_O_2_ content

The last fully expanded leaf from three plants per treatment were taken for the measurements as three replications. The absorbance was detected at 560 nm using a UV spectrophotometer (TU-1810, Beijing purkinje general instrument, Beijing, China) using ferrous oxidation xylenol orange assay. In detail, 2 g leaf sample was grounded at 4 °C in 2 mL pre-cool acetone and centrifuged at 10,000 r min^− 1^ at 4 °C for 10 min. Then, 1 mL supernatant was well mixed with 2.25 mL carbon tetrachloride and 0.75 mL trichloromethane. Afterwards, 5 mL ddH_2_O was added, well mixed and centrifuged at 5000 r min^− 1^ for 1 min. After that, 1 mL supernatant was well mixed with 2 mL mixture of reagent A and B (1:10, volume ratio). The reagent A contained 3.3 mmol L^− 1^ ferrous sulfate, 3.3 mmol L^− 1^ ammonia sulfate and 412.5 mmol L^− 1^ sulfuric acid. The reagent B contained 165 μmol L^− 1^ xylenol orange and 165 mmol L^− 1^ sorbitol.

### Leaf phytohormone content

The last fully expanded leaf from the top were collected with three replications and immediately frozen in liquid nitrogen for the measurements of ABA, IAA, GA_3_, ZR and melatonin content using ELISA technique. As described by He (1993) [45] and Yang et al. (2001) [46], 2 mL pre-cold 80% (v/v) methanol with 1 mmol L^− 1^ BHT (butylated hydroxytoluene) were added. The samples were homogenized and centrifuged at 10000 r min^− 1^ at 4 °C. Afterwards, the supernatant were passed through a C-18 solid phase extraction column (Waters, Milford, MA) and dried in N_2_. The samples were dissolved in 0.01 mol L^− 1^ PBS (pH 7.4) to determine the levels of phytohormone content. By adding known amounts of standard hormone, the percentage recovery of the ABA, IAA, GA_3_ and ZR were calculated. The samples for melatonin content was homogenized and determined according to the plant MT ELISA kit (Lanpai Bio, Shanghai, China). In detail, the standard and samples were added in the wells, after which 100 μL HRP horseradish peroxidase were added. The samples were covered by adhesive strip and incubated for 1 h at 37 °C. The wells were aspirated and washed, which was repeated five times. Subsequently 50 μL substrate A and 50 μL substrate B were added to the wells, which were incubated for 15 min at 37 °C in darkness. Afterwards, 50 μL stop buffer was added to the wells. The optical density (O.D.) at 450 nm was recorded using a microtiter plate reader within 15 min. The melatonin content in the samples was calculated by comparing the O.D. of the samples from a standard curve.

### Leaf chlorophyll fluorescence

The last fully expanded leaf from the top of four plants per treatment were used for the measurements after 42 h treatment. The seedlings were put in darkness for 20 min before quenching analysis. The F_v_/F_m_, F_q_^′^/F_m_^′^, q_L_ and NPQ under a PPFD of 285 μmol m^− 2^ s^− 1^ with internal light source were measured using MINI-PAM-II (Walz, Eiffeltrich, Germany) and calculated according to the summary by Murchie and Lawson (2013) [47]. Rapid light-response curves of ETR were obtained by exposing the leaf sample for 30 s to each of 10 increasing actinic light levels from 0 to 1496 μmol m^− 2^ s^− 1^ PPFD (0, 24, 45, 65, 90, 126, 190, 285, 420, 630 and 822 μmol m^− 2^ s^− 1^) using MINI-PAM-II. The main vein of the leaf was avoided during the measurements.

### Library construction and RNA-seq of leaf samples

The first fully expanded leaf from the top of the plants was collected with three replicates after 42 h per treatment. The 12 samples were immediately frozen in liquid nitrogen and stored at − 80 °C before sequencing. Total RNA was extracted using the Total Plant RNA Extraction Kit (Karroten, Beijing, China). The RNA concentration and integrity of the RNA was detected using NanoDrop 2000 (Thermo Scientific, DE, USA) and RNA Nano 6000 of Agilent Bioanalyzer 2100 system (Agilent Technologies, CA, USA) to ensure the qualified sample for RNA-seq. The libraries were constructed using NEBNext UltraTM RNA Library Prep Kit for Illumina (NEB, USA). In detail, the mRNA was captured poly-T oligo-attached magnetic beads (NEB, USA). The mRNAs were randomly fragmented using NEBNext First Strand Synthesis Reaction Buffer (NEB, USA). The first strand of cDNA was synthesized using random hexamers and the second strand of cDNA was synthesized by adding buffer, dNTPs, RNase H and DNA polymerase I with the mRNA as a template. The cDNAs were purified using AMPure XP system (Beckman Coulter, Beverly, USA), after which the distal ends were repaired and the tails as well as adapters were linked. To select the fragment size, the AMPure XP beads were used and cDNA library was obtained by PCR enrichment.

The quality of the cDNA library were determined using Agilent Bioanalyzer 2100 (Agilent Technologies, Palo Alto, CA) and qPCR (BioMarker Technologies Co. Ltd., Beijing) to make sure the cDNA library was qualified for sequencing. Then, the qualified cDNA library was sequenced using the HiSeq X-ten (BioMarker Technologies Co. Ltd., Beijing).

### Identification of differentially expressed genes (DEGs) and functional annotation in leaf of tomato ‘Jinlingmeiyu’

RNA-Seq data for samples of plants treated with the four treatments were obtained from three biological replicates. Empty reads, adapter sequences and low-quality sequences were removed from raw reads to obtain clean reads. The transcript abundances of genes in the samples were calculated by fragments per kilobase of exon per million fragments mapped (FPKM). The DEGs in tomatoes in response to the four treatments were obtained using bioinformatics methods. The criteria for DEGs selection was fold change ≥2 and false discovery rate (FDR) < 0.05. The function of the DEGs were conducted based on GO and KEGG database.

### qRT-PCR validation

Total RNAs were extracted using Trizol reagent (Invitrogen, CA, USA). Using Prime Script RT reagent Kit (TaKaRa, Dalian, China), the qualified total RNAs were reverse transcribed to cDNA at 42 °C for 60 min, 70 °C for 15 min and kept on ice for 3 min in an Eppendorf Mastercycler Gradient (Mastercycler®ep realplex, Hamburg, Germany). Using SYBR Premix Ex TaqTM (Takara, Dalian, China), the cDNA was amplified at 95 °C for 2 min, 40 cycles of 95 °C for 15 s, 60 °C for 15 s and 72 °C for 20 s in an Eppendorf real-time PCR machine (Mastercycler®ep realplex, Hamburg, Germany). The reactions were repeated three times as three technical repetitions with three biological repetitions. The expression levels were calculated by the 2^−ΔΔCt^ method with *EF1α* as reference gene. The primers for qRT-PCR were designed using primer 5.0 (Primer-E Ltd., Plymouth, UK) shown in Additional file 3: Table S7.

### Data analysis and data access

Analysis of variance (ANOVA) between the physiological parameters of plants at the control, cold, drought and combined stress were performed using SPSS 16.0 (SPSS Inc. Chicago, IL, USA). The qRT-PCR data of the 12 genes in tomatoes at the four treatments were analyzed by ANOVA. The KEGG pathway enrichment analysis of the DEGs was performed using hypergeometric distribution (BioMarker Technologies Co. Ltd., Beijing). The significant differences were considered at *P* < 0.05. The sequencing data has been submitted to NCBI under the accession number of SRP156535 at https://www.ncbi.nlm.nih.gov/sra/SRP156535.

## Additional files

Additional file 1: **Figure S1.** (**A**) Number of new genes predicted by de novo assembly, (**B**) distribution of Nr homologous species and (**C**) eggNOG function classification of consensus sequence. The Nr indicated non-redundant protein sequence database, the website of which was ftp://ftp.ncbi.nih.gov/blast/db/. The eggNOG indicated evolutionary genealogy of genes: Non-supervised Orthologous Groups, the website of which was http://eggnog.embl.de/. (JPG 158 kb)

Additional file 2: **Figure S2.** The KEGG (Kyoto Encyclopedia of Genes and Genomes, http://www.genome.jp/kegg) pathway enrichment analysis of the DEGs (differentially expressed genes) between (**A**) control vs cold, (**B**) control vs drought, (**C**) control vs combined stress and (**D**) cold vs drought. (JPG 230 kb)

Additional file 3: **Table S1.** Reads overview of the RNA-seq data in tomato at control, cold, drought and their combination**. Table S2.** The detailed information of the 12 differentially expressed genes (DEGs) for qRT-PCR validation. **Table S3.** Differentially expressed genes (DEGs) involved in reactive oxygen species (ROS) in tomato at control, cold, drought and their combination. **Table S4.** Differentially expressed genes (DEGs) involved in signal transduction in tomato at control, cold, drought and their combination. **Table S5.** Differentially expressed genes (DEGs) involved in phytohormone signal transduction in tomato at control, cold, drought and their combination. **Table S6.** Differentially expressed genes (DEGs) involved in photosynthetic electron transport in tomato at control, cold, drought and their combination. **Table S7.** Primers for qRT-PCR validation of differentially expressed genes (DEGs). (XLSX 714 kb)

## Data Availability

The sequencing data has been submitted to NCBI under the accession number of SRP156535 at https://www.ncbi.nlm.nih.gov/sra/SRP156535. The seeds, plant materials and datasets during or analyzed during the current study available from the corresponding author on reasonable request.

## Abbreviations

ABA: abscisic acid
DEGs: differentially expressed genes
DW: dry weight
ETR: electron transport rate
FDR: false discovery rate
FPKM: fragments per kilobase of exon per million fragments mapped
F_q_^′^/F_m_^′^: quantum yield of photosystem II
F_v_/F_m_: maximum potential quantum efficiency of photosystem II
FW: fresh weight
GA_3_: gibberellin
GO: gene ontology consortium
IAA: auxin
KEGG: kyoto encyclopedia of genes and genomes
NPQ: non-photochemical quenching
PPFD: photosynthetic photon flux density
PSII: photosystem II
q_L_: fraction of open PSII centers
qRT-PCR: quantitative real-time PCR
RWC: relative water content
TW: turgid weight
ZR: zeatin riboside
