# Supplementary figures and images for: Physiological analysis and transcriptome sequencing reveal the effects of combined cold and drought on tomato leaf
Source: BMC Plant Biol. 2019 Aug 27;19:377. doi: 10.1186/s12870-019-1982-9 (PMC6712725; doi:10.1186/s12870-019-1982-9)

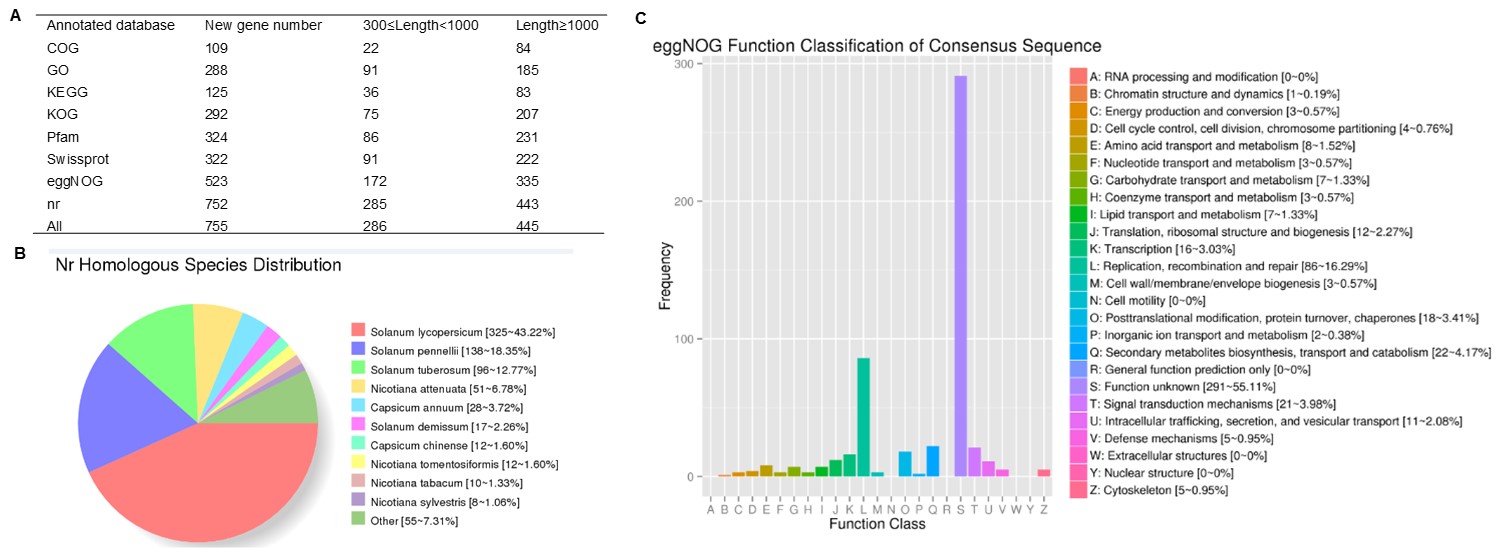

Supplement: Supplementary file 1 — Figure S1. (A) Number of new genes predicted by de novo assembly, (B) distribution of Nr homologous species and (C) eggNOG function classification of consensus sequence. The Nr indicated non-redundant protein sequence database, the website of which was ftp://ftp.ncbi.nih.gov/blast/db/. The eggNOG indicated evolutionary genealogy of genes: Non-supervised Orthologous Groups, the website of which was http://eggnog.embl.de/. (JPG 158 kb) [file 12870_2019_1982_MOESM1_ESM.jpg]

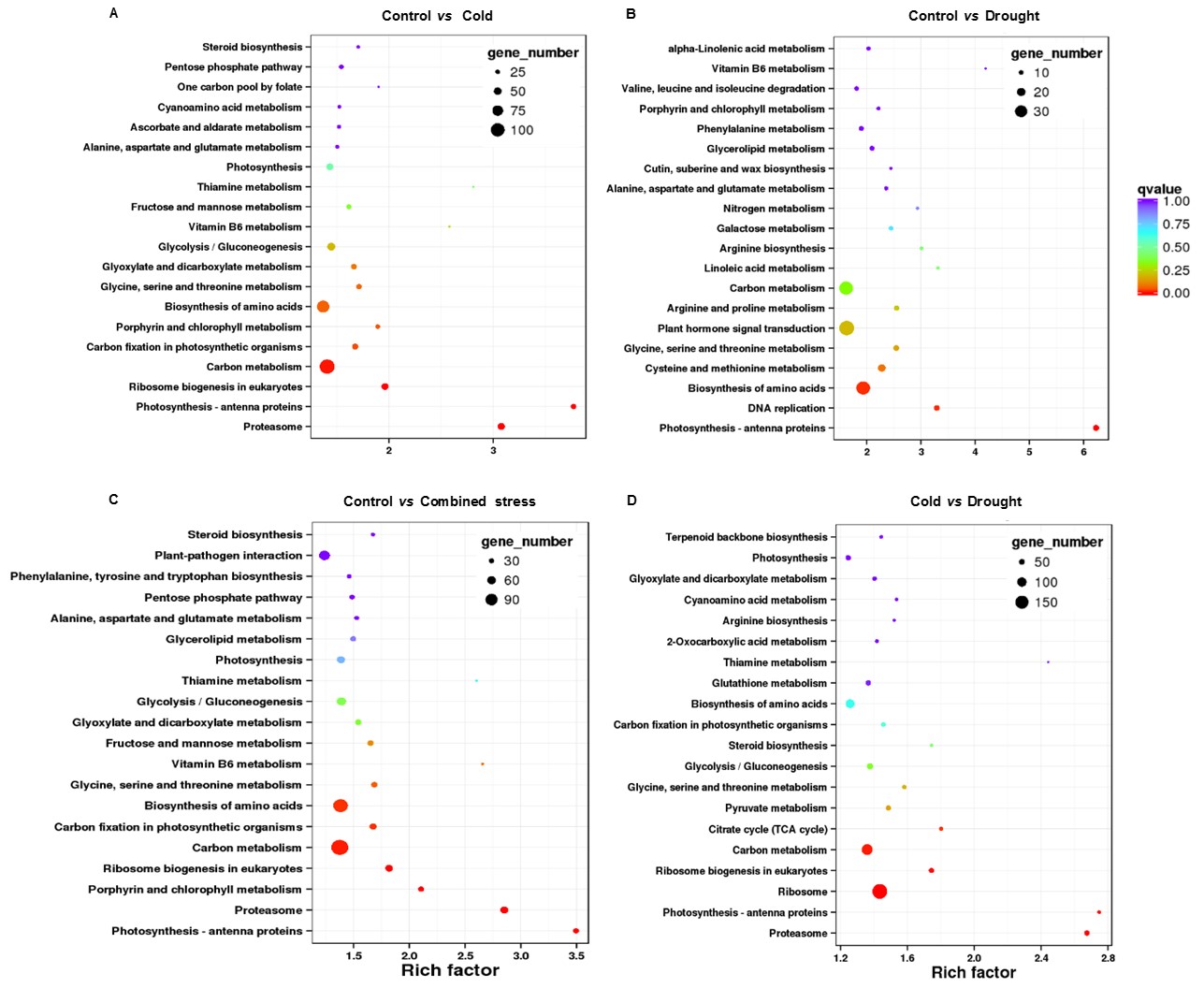

Supplement: Supplementary file 2 — Figure S2. The KEGG (Kyoto Encyclopedia of Genes and Genomes, http://www.genome.jp/kegg) pathway enrichment analysis of the DEGs (differentially expressed genes) between (A) control vs cold, (B) control vs drought, (C) control vs combined stress and (D) cold vs drought. (JPG 230 kb) [file 12870_2019_1982_MOESM2_ESM.jpg]
